# Implementation-effectiveness trial of an ecological intervention for physical activity in ethnically diverse low income senior centers

**DOI:** 10.1186/s12889-017-4584-1

**Published:** 2017-07-18

**Authors:** Porchia Rich, Gregory A. Aarons, Michelle Takemoto, Veronica Cardenas, Katie Crist, Khalisa Bolling, Brittany Lewars, Cynthia Castro Sweet, Loki Natarajan, Yuyan Shi, Kelsie M. Full, Eileen Johnson, Dori E. Rosenberg, Melicia Whitt-Glover, Bess Marcus, Jacqueline Kerr

**Affiliations:** 1 Department of Family Medicine and Public Health, University of California, 9500 Gilman Drive, San Diego, La Jolla, California, 92093-0811 USA; 2 Department of Psychiatry, University of California, San Diego, La Jolla, California, USA; 3 Moores Cancer Center, University of California, San Diego, La Jolla, California, USA; 4 Omada Health, Inc, 500 Sansome Street, San Francisco, California, USA; 5 Group Health Research Institute, Kaiser Permanente, Seattle, Washington USA; 6 Gramercy Research Group, Winston-Salem, North Carolina USA

**Keywords:** Peer led, Older adults, Randomized control trial, Walking, Cost-effectiveness, Low-income

## Abstract

**Background:**

As the US population ages, there is an increasing need for evidence based, peer-led physical activity programs, particularly in ethnically diverse, low income senior centers where access is limited.

**Methods/design:**

The Peer Empowerment Program 4 Physical Activity’ (PEP4PA) is a hybrid Type II implementation-effectiveness trial that is a peer-led physical activity (PA) intervention based on the ecological model of behavior change. The initial phase is a cluster randomized control trial randomized to either a peer-led PA intervention or usual center programming. After 18 months, the intervention sites are further randomized to continued support or no support for another 6 months. This study will be conducted at twelve senior centers in San Diego County in low income, diverse communities. In the intervention sites, 24 peer health coaches and 408 adults, aged 50 years and older, are invited to participate. Peer health coaches receive training and support and utilize a tablet computer for delivery and tracking. There are several levels of intervention. Individual components include pedometers, step goals, counseling, and feedback charts. Interpersonal components include group walks, group sharing and health tips, and monthly celebrations. Community components include review of PA resources, walkability audit, sustainability plan, and streetscape improvements. The primary outcome of interest is intensity and location of PA minutes per day, measured every 6 months by wrist and hip accelerometers and GPS devices. Secondary outcomes include blood pressure, physical, cognitive, and emotional functioning. Implementation measures include appropriateness & acceptability (perceived and actual fit), adoption & penetration (reach), fidelity (quantity & quality of intervention delivered), acceptability (satisfaction), costs, and sustainability.

**Discussion:**

Using a peer led implementation strategy to deliver a multi-level community based PA program can enhance program adoption, implementation, and sustainment.

**Trial registration:**

ClinicalTrials.gov, USA (NCT02405325). Date of registration, March 20, 2015. This website also contains all items from the World Health Organization Trial Registration Data Set.

## Background

Older adults (aged 65+) will comprise 22% of the population by 2030 with ethnically diverse seniors making up 28% of that total [1]. Less than 3% of older adults are meeting physical activity (PA) guidelines when objectively measured by accelerometer, despite well documented health benefits of PA [2]. Accelerometer studies in older adult populations have also shown disparities in PA by race and income [3, 4] and there are well documented disparities in environments and resources available for PA [5]. Given the burden of inactive older adults on the healthcare system, and the relatively low costs of PA interventions, it is imperative that a greater number of older adults have access to efficacious, evidence based PA programs and neighborhoods that support PA [6].

The Surgeon General’s 2015 Call to Action to Promote Walking and Walkable Communities encourages more older adults to be physically active through walking, and identifies the need for walkable communities to support this activity [7]. Walking as the physical activity target was promoted because of its beneficial effects for individuals and communities. For example, individuals who walk become healthier and communities that support walking through programs, policies, and planning are more cohesive and livable [7]. The Call to Action identifies the need for more research on the impact of how multi-level interventions work, evaluation of the continued efficacy, effective implementation and sustainability of existing or new programs, and economic analysis of the costs and benefits of PA programs and interventions. The National Institutes of Health is also supporting more research into multi-level interventions that are hypothesized to be more scalable and sustainable [8] and is advancing a more scientific approach to dissemination and implementation studies to assess and enhance the effectiveness and sustainability of evidence based interventions [9]. These initiatives and the Surgeon General’s Call emphasize the importance of community settings with organizational infrastructure, financial support, and community reach for program delivery.

We developed a multilevel walking intervention targeting diverse and low income older adults in senior centers that aligns well with the Surgeon General’s Call. It is currently being implemented in a cluster randomized controlled trial using a hybrid implementation-effectiveness design [10] by trained peer health coaches. The evaluation includes objective assessment of behaviors and clinical outcomes as well as measuring intervention costs and individual and organizational predictors of implementation success and sustainability. Our objective is to describe the intervention protocol and study design for the program which is called ‘Peer Empowerment Program 4 Physical Activity’ (PEP4PA).

## Methods

### Study design

**Fig. 1:**
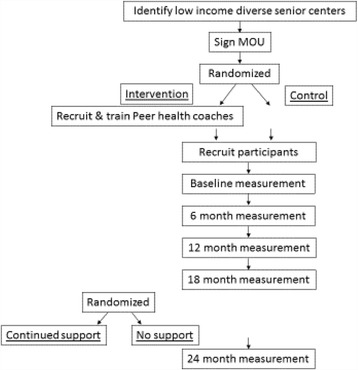
Study design and procedural flow

This study is a two year, two arm, cluster randomized *effectiveness-implementation hybrid type I1 design* that involves testing a clinical intervention while gathering information on implementation strategies and outcomes [10]. This design includes equal attention to clinical outcomes and metrics of program delivery, fidelity, and the processes which can affect program delivery and success. The design and flow of the study is outlined in Fig. 1.

Half of the 12 senior centers will be randomized to the PEP4PA intervention and half to the control condition (usual care). We will recruit 24 peer health coaches, (approximately 4 peer health coaches per intervention center), who will be trained to deliver and sustain the PA intervention. We then recruit 408 older adult participants in the 12 senior centers (approximately 34 participants per center) and. Center level randomization is necessary to avoid cross-contamination as the intervention involves senior center staff and changes to the environment in and around the senior centers. We are comparing the PEP4PA intervention to a usual care control condition because 1) usual care within senior centers often includes planned programing for PA and social and educational classes and events, 2) we aim to examine the cost-effectiveness of the PEP4PA program compared to usual programing, 3) it is important to have a control condition when studying implementation strategies, and 4) older adults’ PA naturally declines over time, so studying that rate in usual care is important. The trial is 24 months. We hypothesize that PEP4PA will be more effective than usual care for increasing and sustaining PA because of the empowering multilevel elements included in PEP4PA (see content below).

To assess whether the program is sustained when study investigator support is removed, we are utilizing a second randomization after 18 months in the 6 centers that received PEP4PA. Half the intervention centers will be randomized to receive no further support from the study staff, half to continue receiving support. Continued support includes remuneration of peer health coaches, monitoring and feedback related to program delivery goals, and identification and support for continued training needs. IRB approval for the study was obtained from the University of California, San Diego Human Research Protections Program (#150336). The study investigators held regular meetings to check the progress of preparing, tracking, and updating the progress on implementing the PEP4PA intervention study on a biweekly basis from January 2015. A community advisory board meets quarterly to provide advice on recruitment, training and sustainment. A formal data monitoring committee was not needed in this trial as it has known minimal risks to participants. No concomitant care or interventions were prohibited during the trial.

### Randomization

Randomization is assigned at the senior center level. Both arms of the study are described to the center director and randomization occurs once centers sign a memorandum of understanding agreeing to either condition. The study statistician developed a randomization table that includes 12 spots with masked group assignments and is password protected. The program manager (who does not interact with the center) adds centers to the table in the order in which they are enrolled and unmasks the group assignment. The center assignment is then relayed to the study personnel who notify the center of their condition. Peer health coaches in the intervention centers are then recruited and trained in the intervention components. Participants are recruited to the specific study condition. While this may lead to self-selection bias, this process is typical of pragmatic trials. Further, baseline differences in groups will be adjusted for in analyses. A permuted block design ensures equal numbers of centers are in the intervention and control condition. At 18 months, the same procedures are employed to randomize the 6 intervention centers to ‘continued funding and study staff support’ or ‘no support’. The statistician will be blinded to the intervention assignment. Data collectors will not be blind to the study condition as the intervention includes on site promotional materials that they may observe. Further, participants tend to share information with staff on their walking and study activities. Given that the outcomes involve objective measures, rather than rater judgments, this is considered acceptable.

### Recruitment and eligibility criteria

We identified senior and community centers that are in communities serving a low-income population in San Diego County. Centers in low income communities are identified by the average annual household income of the census tract around the center’s address. Centers in census tracts with annual household income below San Diego County median are considered low income. We contact all centers, either by phone or in person, to obtain basic information on their size and program offerings. Confirm the number of older adults they serve, classes offered, hours, and space available. Interested centers then fill out an application verifying eligibility criteria including: at least 1 physical activity class offered, able to recruit 35 participants and, in intervention sites, can recruit 4 health coaches and a staff member, have sufficient space for study activities and can commit to the 2 year study period. This application and a memorandum of understanding is signed so that details of the nature of the study and commitments are clear. Centers that meet the criteria are then selected and offered to participate in the study. After being randomized into either intervention or control, the center directors identify at least one staff member to attend the training and assist with the implementation and logistical coordination of the intervention at the institutional level. This staff person recommends potential peer health coaches who then meet with UCSD staff to learn about the role and complete an interview, if interested. The peer health coach role is also announced in the surrounding community and networks. No specific educational prerequisites are required for the peer health coaches. A willingness to lead and experience with teaching, coaching, or community advocacy are desired. Participant recruitment in the centers occurs through word of mouth, flyers, information tables, social media and presentations. To recruit residents who may not normally attend the center, we place advertisements in local publications and media, at churches, and in senior housing complexes. We also use marketing company data to identify participants meeting the age criteria (50+), with an address within 2 miles of the center. The marketing company uses public data sources such as census data, survey reported data, and the white pages. People who meet the age criteria receive a letter from the study team explaining the opportunity to participate in the study and the date and location of a study recruitment event. Participants are recruited primarily when the study starts at each site, but senior center members can enroll on a rolling basis at any time up to 12 months from baseline. Participant recruitment began in 2015.

Older adults, 50 years and over, who are eligible according to our criteria are consented, enrolled and assessed. Eligibility criteria include: a) completing a Timed Up and Go Walk test [11] within 30 s, with a walking aid if needed, b) not having had a fall resulting in a hospitalization during the previous 12 months, c) able to walk without human assistance (cane or walker use okay), d) able to provide informed written consent, e) able to complete surveys without assistance, f) able to read and write in English or Spanish, g) able to complete study activities and wear devices, and h) complete a post-consent review comprehension test to assess comprehension of their role as a participant in the study.

### PEP4PA intervention

**Fig. 2:**
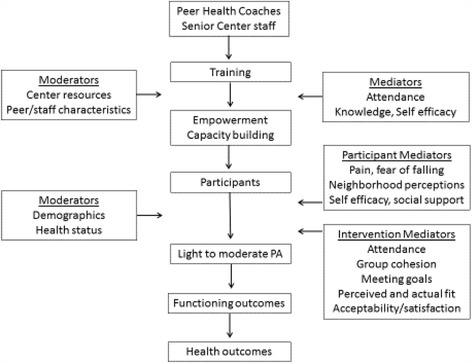
Conceptual model of intervention delivery, mediators and moderators

The Social Ecological Model provides a framework for addressing behavior change at different levels of influence, from the individual to policy [12]. Within each level, Social cognitive theory (SCT) provides guidance for specific behavior change strategies. We are unique in applying these behavior strategies consistently across the individual, interpersonal and institutional levels of the Ecological Model. PEP4PA employs strategies from SCT identified by Michie et al. as having most impact on behavior change [13]. These include goal setting, self-monitoring, feedback, positive reinforcement, social support, planning, and problem solving. Such individual behavior change strategies are typically not part of existing group-based PA programs in senior centers. Figure 2 outlines how theory informs the intervention delivery and evaluation.

PEP4PA also draws upon Empowerment theory which has not been used in previous PA research with older adults. Empowerment theory posits a mechanism for community participation, enhancing community problem-solving skills, providing leadership training, and creating jobs in the community [14]. Strategies to facilitate empowerment include enhancing experience and competence, enhancing group structure and capacity, enhancing environmental support and resources, and removing social and environmental barriers [15] Organizational empowerment includes opportunities for members to take on meaningful and multiple roles, and a peer-based support system that helps members develop a social identity [16]. Mentoring, supportive peer relationships, and a political consciousness have also been identified as mechanisms [17]. PEP4PA draws upon Empowerment theory and the intervention strategies and goals mirror its key elements. PEP4PA trains peer health coaches and senior center staff to improve their own health awareness, social support skills, organizational skills, and their ability to advocate and build capacity within the senior centers. Peer health coaches take on a mentoring role for study participants and encourage members to help them run the program. Involving members in new program development can lead to empowerment. Peer health coaches and staff receive specific training in advocating for walkable communities from our community partner Circulate San Diego, including identifying barriers and prioritizing improvement projects. PEP4PA aims for the program to become institutionalized which is also a feature of Empowerment Theory [17]. Success stories are also a mechanism for realizing community empowerment [17]. PEP4PA includes sharing of individual, group and community successes in discussions, events, photos, and video testimonials.

Peer health coaches receive 10 h of basic training (2.5 h a day over 4 days) prior to working with program participants. Ten modules are covered during the training time as outlined below. Each training day has specific objectives that are described at the beginning of the training and then revisited at the end to ensure they have been met. Self-reported confidence to lead each of the components is assessed at the end of the training and additional attention is given to the items identified as low confidence. The training was pilot tested with 4 peer health coaches who led an 8-week pilot program in a senior center. Improvements to the content and design were made before being implemented in the current trial. The training is delivered by an experienced health educator and group facilitator with a Master in Public Health and several years of experience in health promotion research (Table 1).

**Table 1:** Training content and objectives

Day	Topic	Learning Objectives
Day 1	Module 1: Laying the Foundation	1. Explain: • *Why:* Significance of the Program • *What:* Goal of the Program • *How:* Program Components • *Who:* Roles • *When:* Timeline 2. Understand what elements in the environment contribute to people’s ability to be active • Understand the importance of building partnerships to enhance and sustain the program
	Module 2: Community Project, Partnerships and Sustainability	
Day 2	Module 3: Individual Coaching	1. Demonstrate how to use 3 self-monitoring tools for individual coaching 2. Explain the importance of establishing a baseline/starting point before setting a goal 3. Explain the Short Term & Long term step goals for the program 4. Demonstrate the ability to complete the 7 individual coaching steps 5. Describe important leader qualities when coaching
Day 3	Module 4: Group Sharing	1. Understand key skills to facilitate the group sharing 2. Locate the health tips 3. Describe what needs to be completed before, during, and after leading a walk 4. Describe what to do during each crosswalk signal and what hazards to keep an eye out for 5. Understand Adverse Event/Incident Reporting Procedures
	Module 5: Health Tips	
	Module 6: Group Walk	
	Module 7: Adverse Event/Incident Reporting	
Day 4	Module 8: Having Fun, Celebrating Success & Rewards	1. Understand the importance of fun & celebration in keeping the group motivated & engaged 2. Understand the importance of evaluation & tracking 3. Explain which types of tracking & evaluating will be part of peer health coach role 4. Understand how to use the tablet for tracking & evaluating tasks
	Module 9: Evaluation & Tracking	
	Module 10: Staying Organized & Planning Ahead	

After completion of the basic training, the peer health coaches participate in 6 h of practice training (2 h/3 days). This allows time for the peer health coaches to practice leading each of the program components (i.e., group walks, health tips, group sharing, and individual coaching) and get feedback from other peer health coaches and from the study staff training leader.

Once the peer health coaches have completed the 16 h of training, they begin leading the group of participants with supervision from the study staff. They are observed & evaluated with a clear set of standards. Study staff meets with them one on one to review the evaluation & provide feedback on needed improvements. Once they have met all of the standards for each of the program components, they are certified as a peer health coach. After being certified, they are able to lead on their own without the presence of study staff. However, quality checks are administered monthly after the first 3 months.

### Intervention components

Individual level - Support at the individual level includes: printed materials, self-monitoring, pedometers, progress charts, and tailored coaching.

Participants are supported to engage in the behavioral strategies outlined below to increase their walking. The long-term goal is a 2000 daily step increase over individual baseline levels, achieved through the activities outlined below. A goal of 2000 extra daily steps was selected because it is appropriate for older adults and provides significant health benefits [18]. Steps are also easily measured by a pedometer, a simple tool for older adults to use for self-monitoring that has been shown to be successful in intervention trials. All participants receive the same goal, regardless of their individual starting point, which adds to the group cohesion. Further, the goal can be achieved gradually over 5 months with a 100 daily step increase per week. This schedule is easy to communicate and can be re-started by participants after setbacks, such as illness, to get them back on track (Table 2).

**Table 2:** Behavior strategies for individual participants

Monitoring/evaluation	Pedometers to monitor steps & charts to show progress
Goal setting	2000 daily step increase over baseline levels, achieved through individual walking & group walks
Feedback	Graphs of daily steps over time. Review of progress & verbal positive reinforcement from peer health coaches bi-weekly.
Rewards/Recognition	Monthly group celebrations and individual recognition
Social support	Peer health coach & staff encouragement. Group walks at appropriate levels and other group activities/events led by peer health coaches. Group sharing of challenges/solutions.
Role models/success stories	Peer health coaches model step goals. Participants hear about others who meet goals/overcome challenges during group sharing.
Positive experience	Fun events & supportive atmosphere in center. Well organized walks with different abilities accounted for. SMART goals that are challenging but achievable. Clear short and long term goals i.e. 100/day extra each week to meet 2000/day step increase as a long term goal.
Cues/reminders	Phone calls or e-mails from peer health coaches to remind participant to attend center events, in person check ins & logs for steps. Promotional materials in center.
Planning/scheduling	Planning attendance at events, reviewing steps across day with pedometer, planning when to get steps.
Problem solving	Work on overcoming barriers with peer health coaches. Learn how others overcome barriers. Reduction of environmental barriers e.g. crossing times, lack of equipment through community projects led by peer health coaches.
Relapse prevention	100 step goal schedule for returning to normal after illness. Set goals for how to maintain step increases. Continued monitoring from pedometer and peer health coaches.

#### Printed materials

At the beginning of the program, participants receive a folder of tip sheets (i.e. walking safety, overcoming barriers, social support, rolling with relapse) that pertain to the health tips led each week by the peer health coaches. They also receive a copy of the book *Exercise & Physical Activity: Your Everyday Guide* from the National Institute on Aging [19].

#### Self-monitoring

Participants monitor their steps daily using a pedometer & step log. The step logs have carbon copies so participants can keep a copy and turn in a copy to their peer health coaches. They turn in their step logs weekly to their peer health coach who provides them with a progress chart bi-weekly. The progress chart shows a weekly average of steps walked, their weekly step goal and a projected progress line so they can stay on track with working towards their long term goal of 2000 additional steps from their baseline.

#### Tailored coaching

Participants meet one on one with their peer health coach at the beginning of the program to build rapport and receive help with initial goal setting. The peer health coaches prompt the participants to identify & record their motivation for being active, what barriers they anticipate getting in the way of reaching their goals, solutions that will allow them to overcome the anticipated barriers, and who in their life can serve as a social supporter to encourage them to reach their walking goals. The peer health coaches then guide them through seven individual coaching steps, 1) Health check-in, 2) Exploration of why goals were met or not met, 3) Set goal for next week, 4) Create an action plan, 5) Assess confidence, 6) Remind them of next study event, and 7) Review- participant repeats back. The peer health coaches monitor the participant’s progress throughout the program using their step logs and provide additional goal setting support as needed. If a participant is reaching their steps on 4 or more days, they are encouraged to raise their daily step goal by 100 additional steps for the next week. If a participant is reaching their step goals on 4 or more days, but exceeding the goal by 500 additional steps they are reminded of pacing and the peer health coach checks to ensure they are not incurring any overuse injuries before encouraging them to raise their steps by an additional 100 steps. If a participant is not meeting their step goal on 4 or more days, they are encouraged to try for the same goal or reduce if needed, after discussing barriers to reaching their goal.

#### Interpersonal level

Participants meet as a group twice per week for group walks. Preceding the walk, the peer health coaches lead the group sharing or health tip session. Social support is integrated through group based health tips and group sharing.

#### Health tips

Once a week before going on the group walks, the peer health coaches deliver a health tip. The health tips are aimed at increasing participants’ knowledge around how to safely & gradually increase their walking. After the first 3 months of the program, participants take turns drawing pre-written cards with a health tip and read to the group. This keeps participants engaged in this important part of the program and leads to more group sharing and discussion of health benefits.

#### Group sharing

Once a week before the group walk, the peer health coaches facilitate a group sharing. This is a time to receive support from others in the group who are working on a common goal, to share stories of success to inspire others, and to share new strategies for achieving steps. The coaches ask four questions to engage the group around their shared experience of trying to increase their steps. 1) Are there any challenges you have been facing in trying to reach your walking goal? 2) Any new benefits you have experienced since you have been walking more? 3) Any new strategies or walking routes you have discovered to help reach your goal? 4) Any relevant articles in the news or events in the community related to walking?

#### Community level

Peer health coaches lead the group in a community project and work to build community partnerships to help enhance & sustain the program.

#### Community project

A local pedestrian advocacy organization, Circulate San Diego, leads a presentation on walkability at the 4 month timepoint, engaging participants in a discussion around what elements in the environment contribute to their ability to walk safely. The group is guided on a walk audit to identify barriers to walking in the community surrounding the center. The participants are then tasked with prioritizing which issues are the most impactful for increasing walking. The group is further trained on how to effectively advocate for changes relative to the project they have chosen. They are provided guidance around how to create an action plan and the peer health coaches take the lead to ensure the action plan is executed. Currently, one site is working on getting the city to put in a new crosswalk at a busy intersection near the senior center that would connect it to a bus line. Another site is working on repairing the pavement around a park next to the senior center, which is a site that they walk the most. Another site is working on repairing the sidewalk in an area that had a tripping hazard. Other example community projects that were successful in our previous work in retirement communities included street and access route clean ups from debris and vegetation, improvements to crossings with auditory and visual timers and longer crossing times, and parking structure re-design so cars did not obstruct the sidewalk [20].

#### Community partnerships

One goal of the program is to identify ways to sustain the program in the centers once the study is over. The peer health coaches are encouraged to develop community partnerships during the weeklong basic training. They complete an asset mapping activity where they identify businesses, schools and organizations that reside within the surrounding community and think creatively around ways they might involve these groups to help enhance or sustain the program. This may include financial contributions, in-kind donations, a walking destination or an intergenerational opportunity.

At 6 months, study staff meets with the center directors, staff and Peer health coaches to start to develop a sustainability plan. The sustainability plan is tailored to each site factoring in a number of center characteristics (e.g., funding, staffing structure). During the first meeting, all of this information is gathered to help guide the discussion. For some sites, program sustainment may include a reallocation of existing funds while for other sites it may involve applying for a new grant or seeking out a corporate sponsor. The decision makers are identified early on and efforts to communicate the program’s value are made throughout the program, including invitations to take part in program activities. The peer health coaches and staff are both involved in the sustainability planning process. The sites work to have a plan in place by 18 months, to support the program if they are not randomized to receive continued financial and training support from study staff. This also prepares them for the post-study period.

Peer health coaches receive a $100 per month honorarium. A budget of $1000 per year is provided to intervention sites for program related expenses. These expenses may be used to pay for participants to be transported to offsite locations for group walks, program supplies or materials, to cover fitness instruction costs, PA equipment, prizes for participant achievement, monthly celebrations, or food for group meetings. These funds are managed by the peer health coaches. Intervention sites receive $600 every 6 months for staff costs related to the intervention, as a staff person is designated to assist with the intervention. All intervention sites receive the peer health coach honorarium, program and staff funds through 18 months. Only those sites randomized to receive continued funding in months 18–24 will receive these funds during that time.

Study staff provide on-going training and feedback from the evaluation tool. They follow up when the evaluation tool is not being used or study goals are not being met. They provide technical support for the evaluation tool. Study staff attend weekly meetings in the first 3 months, reducing this to twice a month from month 3–6, and then monthly in 6 months up to 18 months. Study staff lead the meetings for the first 6 months, and then the peer health coaches lead the meetings thereafter. In sites randomized to the no support arm at 18 months, study staff will only be available for technical issues. They will not provide feedback from the tool, they will not follow up on goals not met or if the tool is not being used, and they will not attend meetings or perform any training during this time.

## Control condition

Centers randomized to the control condition do not receive any intervention from study investigators. The PEP4PA intervention is compared to usual care (i.e. normal center programing) at these centers. In an effort to recruit and retain participants and centers, five health related events are organized by study staff in conjunction with the study assessments. These can include a health fair or speaker on wellness/health issues not related to PA. As part of the center selection process, sites must include PA programming. This can include any number of sessions per week and any type of PA. Typical offerings are the Feeling Fit program, Tai Chi or Chair exercises. Both intervention and control centers must offer PA programing. A schedule of programming is collected monthly to verify programming content.

## Measures

The implementation measures draw upon Proctor’s implementation research framework [21], Aarons’ conceptual model of evidence based practice implementation (EPIS) [22], and Damschroder’s consolidating framework for implementation research (CFIR) [23]. Measures include: appropriateness & acceptability (perceived and actual fit), adoption & penetration (reach), fidelity (quantity & quality of intervention delivered), acceptability (satisfaction), costs, and sustainability. Reach is assessed at the center, staff, peer health coach and participant level at the application, enrollment, intervention and continued delivery phases. Factors that may impact reach are demographics, health status, attitude towards and satisfaction with the intervention, center climate and center infrastructure and size. We also assess outer context influences such as funding, policies, leadership and resources. Data are collected through in person semi-structured interviews and self-report surveys at each measurement point (baseline, 6, 12, 18 and 24 months). All peers, designated staff and directors are assessed. A random selection of participants for interview is made, stratified by baseline step count and age. Measures were designed so that the qualitative and the quantitative measures included common themes and tracked over time. The tracking tablet (described below) assesses and maintains the quantity and fidelity of the program delivered. Observations and ongoing training certificates also assess and ensure the quality. In person observations, according to a standard protocol, occur during the training and certification phases and intermittently thereafter. We will use a convergent mixed methods approach to data analyses to merge the results from the quantitative and qualitative data with the purpose of comparing one set of results with the other.

Survey questions and interviews are performed before, during and after the training and program implementation. Measures are completed by center directors, center staff, peer health coaches and participants to triangulate the factors affecting the implementation process. We explore the impact of the program setting, content, deliverers and users.

### Web-based tablet

A web based tablet database tool was developed for the peer health coaches to track all program activities (i.e. attendance, any modifications to the program, step logs). This provides a measure of program fidelity regarding quantity of intervention delivered. Each coach is given a tablet to track activities while in the field (on a group walk, etc.) Study staff are able to monitor activities remotely and intervene as needed. In addition to the tracking function, the web database serves as a feedback mechanism on the participant, peer health coach, and center level. Peer health coaches can see whether their participants are attending program activities and their progress toward their step goals from the step logs they gather as part of the individual level coaching. The individual coaching steps are included in the database so it gives the coaches a script to follow, while they interact with participants, and auto generates goals based on the data entered. Additionally, it provides goal setting assistance based on whether a participant met their goal on 4 or more days. It also provides feedback on whether they have any coaching tasks that need to be completed (e.g., enter timesheet, enter step logs, schedule individual coaching meeting, etc.) The information tracked through the web database is brought to team meetings between study staff and coaches and guides discussion for program improvements and participant needs. The data are also aggregated so study staff can see how the group is doing as a whole with reaching goals and attending activities. Data stored in the web database, such as the number of members attending, health benefits experienced, or new center members due to the program, can be accessed by the center staff so they can include this information in grants, newsletters, or other dissemination outlets that may aid in sustaining the program.

Measurements include objective measures of PA, sedentary time, sleep, physical functioning, cognitive functioning, height and weight, and blood pressure. Additional surveys are administered at every time point to gather subjective assessment of participants’ depression, stress, quality of life, and correlates of PA such as social support, self-efficacy, pain, and fear of falling.

Study measurement occurs at 5 time points throughout the 24 month study. Study participants are contacted one week prior to scheduled measurement visits or notified in person at center activities. Measurement procedures are separated into two scheduled visits at the center to decrease participant burden. At the first measurement visit, participants are asked to complete the timepoint survey and receive their measurement devices, including a wrist-worn accelerometer and elastic belt with both the hip-worn accelerometer and a GPS device (ActiGraph GT3X+, QStarz Travel Recorder XT). Participants are provided proper wear instructions and asked to wear the hip-worn devices during waking hours and the wrist accelerometer for 24 h a day for the 7 day wear period. Additionally, participants are asked to record their sleep and wake times in a sleep journal for the 7 day wear period. The second measurement visit is scheduled for exactly 1 week (7 days) after the first visit. At the second appointment, participants complete objective assessments of blood pressure, height and weight. At the baseline, 12, and 24 month time points, participants also complete physical and cognitive functioning assessments. At the 6 month and 18 month measurement visits participants are only asked to participate in blood pressure and height and weight assessments, in addition to a shortened study survey. Participants return their measurement devices at the second measurement visit and device data are screened by study staff for valid wear time. Participants who do not have valid device wear (i.e., four days with wear time matching for hip and GPS devices) are asked to re-wear the device for an additional 7 days. Any participants who are not able to attend the scheduled visits are contacted for follow-up and an additional measurement visit is scheduled.

Participants and peer health coaches in the intervention sites receive $10 upon completion of study assessments at 6 and 18 months and $20 at baseline, 12 and 24 month time points. Participants in the control sites are paid the same amount as those in the intervention sites.

## Statistical analysis

We determined sample size for the primary outcome (Aim 1) of improving PA over 12 months. In preliminary analysis of our previous study in retirement communities [24], we observed (i) a mean 40 min/day difference between intervention and control groups for PA at 6 months (SD = 57 min/d), yielding an effect-size of 0.7, and (ii) an intraclass correlation (ICC) of 0.07 for senior center clustering effects on PA (in our pilot study in low-income seniors this ICC < 0.01). Twelve sites and 28 subjects/site will yield 80% power (2-sided test α =0.05) to detect conservative effect-sizes between 0.41 to 0.56 for time-averaged standardized mean differences between arms assuming senior center clustering ICCs ranging from 0.05 to 0.1, and autocorrelations of 0.5 to 0.8 on within-subject repeated measures of PA. Also, under these assumptions, there is 80% power to detect percentages of 20–25% meeting PA guidelines in the intervention vs 5% (based on pilot data) in the control arms at follow-up. To allow for a worst-case attrition rate of 20% by 6 months (our RC retention rate is 91% at 6 months), we aim to recruit 408 participants (12 sites with 34/site).

The primary analysis will use the intent-to-treat principle. The efficacy of the PEP4PA intervention on PA will be assessed comparing the PA intervention group to the usual care control group on minutes/day of PA and % meeting NHANES criteria measured by accelerometry over 6–12 months. A mixed effects regression model will be used with post-intervention PA at 6 & 12 months as the dependent variable and intervention arm (active vs control) as the independent variable, with baseline PA as a covariate. Gaussian link function will be used for the continuous PA outcome; a binomial link will be used for the binary outcome (meeting vs not meeting guidelines). A random effect for site (senior center) and a subject-specific intercept (nested within site) will be added to the model to adjust variance estimates for clustering within site and within individuals over time. Additional covariates such as gender and age, and any factors found to be imbalanced between treatment arms at baseline will be included to examine the impact of covariates on estimated treatment effects. The efficacy of the PEP4PA intervention to improve physical functioning, blood pressure, depressive symptoms & quality of life will be assessed and will compare these outcomes between treatment and control arms using the same approach.

Cost-effective analysis (CEA) is a method measuring the relative efficiency of alternative interventions. It provides information to decision makers to help them maximize the use of scarce resources available to promote PA. CEA, however, has been largely absent in PA promotion interventions in community settings, and even more so with low-income older adult populations. Researchers either have been primarily interested in evaluating the efficacy or effectiveness of interventions, or have lacked the resources to measure the cost of interventions. In addition, many of the existing studies on CEA analysis of PA interventions do not use a standard measure of cost or effectiveness, which hampers the comparison across interventions. For instance, Wang et al. reported the annual cost of bike and pedestrian trails [25] and Sevick et al. measured effectiveness by minutes on a treadmill [26].

In the proposed project, we will use two common measures of cost-effectiveness, cost per MET (metabolic equivalent) hour and cost per QALY (quality adjusted life year). Cost per MET hour captures intervention efficiency in improving PA outcomes, and cost per QALY measures intervention efficiency in promoting the broader concept of quality of life. A MET represents the ratio of energy expended divided by resting energy expenditure. MET hours are derived by multiplying METs associated with the type and intensity of the PA by the time spent on the PA. The type, intensity and time of PA will be derived from the Actigraph 3X–plus model as described above. QALYs will be estimated using the PQOL. The use of common measures of cost-effectiveness will allow us to compare the efficiency of PEP4PA with other PA interventions designed for older adults and help policy makers to prioritize among evidence-based interventions [27]. Cost-effectiveness measures will provide information to help community health leaders, planning groups and senior centers to replicate and disseminate the PEP4PA intervention. Therefore, we will use a senior center perspective, which could also serve as a basis for future CEAs with broader perspectives such as health care system or social perspective.

Intervention costs will be estimated from the study data using standard financial accounting methods. From a senior center perspective, cost will account for all direct resources needed to conduct the intervention. It will not include costs incurred by the intervention participants such as participants’ time during exercise and costs of sports apparel. Costs only related to the assessment of the intervention, e.g. measurement, will be also excluded from the estimate of intervention costs. The CEA will follow well-established guidelines developed by Drummond et al. [28] and Haddix et al. [29] including identification of all relevant costs and consequences for the intervention, accurate measurement in appropriate effectiveness units, sound valuation, and sensitivity analysis to test uncertainties. The final outcome of the CEA is an *incremental cost effectiveness ratio (ICER)*, the ratio of the differences in costs and effectiveness between intervention and control arms [30]. To allow comparisons to other PA interventions, cost per QALY derived from this study will be compared to subjective thresholds of the value of health care ($50,000 per QALY) [30]. We propose to use $1.14 per MET hour cost, the median ICER of community support PA programs in community settings [25], as a tentative cutoff for comparison. All investigators will have access to the final trial dataset.

## Discussion

There are very few rigorous evaluation studies of multi-level interventions for health that include individual, interpersonal, community and policy components. Even fewer include lay professionals who can deliver interventions effectively at low cost in community settings. Evaluations of existing programs in community settings are also lacking or poorly executed [7]. Understanding how to implement and where to promote PA in older adults is imperative to improve quality of life and curb growing health care costs in this population.

PEP4PA is one of the few community based studies that implements a multi-level, peer-led PA intervention for low-income, ethnically diverse older adults. The program could be more sustainable due to the multi-level elements (including community advocacy), training peer health coaches and staff at senior centers to lead the program, and empowerment principles. An additional feature of this study is that it directly assesses sustainability during the study protocol by randomizing community centers to receive either support or no support for the last 6 months. It also uses a hybrid effectiveness-implementation design that allows for assessing the fit of the study to each study site. By evaluating the cost-effectiveness of the intervention, this study will provide useful information for intervention replication and dissemination in community settings.

This study builds upon the MIPARC (Multilevel Intervention for Physical Activity in Retirement Communities) study in retirement communities [31], but adds more in depth training, greater responsibility and empowerment to the peer health coaches, greater staff involvement, and building a sustainability plan from the beginning of the program with input from participants, peer health coaches, staff, and directors. In particular, the peer health coaches are going beyond one time community projects such as cross walk improvements, and going further into leadership roles such as serving on local community advisory boards. We also believe the in-person counseling and simplification of the goal setting will be key to participant success. It is also based in areas of San Diego that have low-income and ethnically diverse seniors who may not have access to quality, sustainable, evidence-based PA programs. Public health is now focusing on implementing and studying programs that are sustainable by their placement and fit in the community. Using the hybrid design allows for understanding both the fit of a program in a center and the way the program is implemented during a research study. The online monitoring through the tablet and interviews at each measurement point allow for fidelity improvements throughout. PEP4PA is a PA program that is designed to be able to translate into existing programming in a senior center. If successful, the next step would be to seek sustainable funding for the program from agencies that are focused on aging. Further, a study on whether this program can be disseminated more widely beyond San Diego County will be warranted.

Challenges to date include the legal requirements for city run senior centers and identifying the proper payment methods for centers or staff to participate in the program. Centers have also closed or had limited hours due to financial difficulties. Preliminary data indicates that roughly 10% of those attending a senior center will enroll in the study. Unfortunately, there are few centers in San Diego County that serve a population of 300+ that would be necessary to meet our goal of 34 participants per site.

In order to reach recruitment targets, we have developed and employed additional community wide recruitment strategies including mailers. We use a marketing list which allows us to identify older adults residing in areas surrounding enrolled centers; a strategy that has been effective in bringing in new members to the centers. If necessary, we will also use ResearchMatch, which is a NIH-sponsored national registry of volunteers who have indicated a willingness to learn more about research studies, to identify people interested in participating that live in the area surrounding the centers. For future sites, all of these methods will be used from the beginning.

We anticipate that participants may drop out given the program lasts for 2 years. We decided that we would enroll participants on a rolling basis so that others may have the opportunity to participate and to ensure the program continues at the centers. Continuous enrollment reflects a pragmatic trial design but presents logistical challenges, including when and how to measure participants and how to account for time in program in analyses. Peers have reported that new participants bring renewed energy to the program.

In summary, this study will advance our understanding of how community members can deliver a multilevel intervention in a community setting.

## Abbreviations

CEA: Cost effectiveness analysis
ICER: Incremental cost effectiveness ratio
IQR: Intraquartile range
MET: Metabolic equivalent
PA: Physical activity
PEP4PA: Peer Empowerment Program 4 Physical Activity
QALY: Quality adjusted life year
